# Consistent responses of soil microbial taxonomic and functional attributes to mercury pollution across China

**DOI:** 10.1186/s40168-018-0572-7

**Published:** 2018-10-18

**Authors:** Yu-Rong Liu, Manuel Delgado-Baquerizo, Li Bi, Jun Zhu, Ji-Zheng He

**Affiliations:** 1 State Key Laboratory of Agricultural Microbiology, Huazhong Agricultural University, Wuhan, 430070 People’s Republic of China; 2 State Key Laboratory of Urban and Regional Ecology, Research Center for Eco-Environmental Sciences, Chinese Academy of Sciences, Beijing, 100085 China; 3 College of Resources and Environment, Huazhong Agricultural University, Wuhan, 430070 China; 4 Cooperative Institute for Research in Environmental Sciences, University of Colorado, Boulder, CO 80309 USA; 5 Departamento de Biología, Geología, Física y Química Inorgánica, Escuela Superior de Ciencias Experimentales y Tecnología, Universidad Rey Juan Carlos, c/Tulipán s/n, 28933 Móstoles, Spain; 6 Faculty of Veterinary and Agricultural Sciences, The University of Melbourne, Parkville, Victoria 3010 Australia

**Keywords:** Soil microbial community, Co-occurrence network, Functional gene, Metagenomics, Mercury pollution

## Abstract

**Background:**

The ecological consequences of mercury (Hg) pollution—one of the major pollutants worldwide—on microbial taxonomic and functional attributes remain poorly understood and largely unexplored. Using soils from two typical Hg-impacted regions across China, here, we evaluated the role of Hg pollution in regulating bacterial abundance, diversity, and co-occurrence network. We also investigated the associations between Hg contents and the relative abundance of microbial functional genes by analyzing the soil metagenomes from a subset of those sites.

**Results:**

We found that soil Hg largely influenced the taxonomic and functional attributes of microbial communities in the two studied regions. In general, Hg pollution was negatively related to bacterial abundance, but positively related to the diversity of bacteria in two separate regions. We also found some consistent associations between soil Hg contents and the community composition of bacteria. For example, soil total Hg content was positively related to the relative abundance of *Firmicutes* and *Bacteroidetes* in both paddy and upland soils. In contrast, the methylmercury (MeHg) concentration was negatively correlated to the relative abundance of *Nitrospirae* in the two types of soils. Increases in soil Hg pollution correlated with drastic changes in the relative abundance of ecological clusters within the co-occurrence network of bacterial communities for the two regions. Using metagenomic data, we were also able to detect the effect of Hg pollution on multiple functional genes relevant to key soil processes such as element cycles and Hg transformations (e.g., methylation and reduction).

**Conclusions:**

Together, our study provides solid evidence that Hg pollution has predictable and significant effects on multiple taxonomic and functional attributes including bacterial abundance, diversity, and the relative abundance of ecological clusters and functional genes. Our results suggest an increase in soil Hg pollution linked to human activities will lead to predictable shifts in the taxonomic and functional attributes in the Hg-impacted areas, with potential implications for sustainable management of agricultural ecosystems and elsewhere.

**Electronic supplementary material:**

The online version of this article (10.1186/s40168-018-0572-7) contains supplementary material, which is available to authorized users.

## Background

Environmental pollution resulting from human activities has a great impact on the biodiversity and functioning of terrestrial and aquatic ecosystems and is a major threat for human health across the globe [1–4]. Importantly, recent studies suggested that changing climate exacerbates global mercury (Hg) pollution by releasing historically stocked Hg in the permafrost [5, 6]. Such a threat is of global concern as Hg can be transported globally [7]. The elevated Hg inputs into environments could have significant effects on soil biodiversity and their associated ecosystem functions [8, 9]. Studies conducted at the local scale seemed to support the idea that Hg pollution can alter soil microbial communities [10–12]. Moreover, previous work based on short-term incubation experiments demonstrated that Hg amendments can alter the soil microbiome and its ecological functions [13, 14]. However, much less is known on the role of Hg pollution in shaping the taxonomic and functional attributes of microbial communities in natural ecosystems across large spatial scale and different types of croplands.

Recent studies suggested that microbial taxa strongly co-occur within ecological network that often are called ecological clusters or modules [15, 16]. Such ecological clusters are expected to have multiple implications for the maintenance of soil fertility, decomposition, and ecological services in terrestrial environments [17–19]. The reason is that different microbial ecological clusters often follow very specific environmental preferences. For example, taxa within some ecological clusters have been found to strongly correlate with low or high pH, mesic or arid ecosystem, and nutrient availability and processes rates [15]. However, despite the importance of these ecological clusters for the understanding of the soil microbiome, their response to long-term field Hg pollution remains largely unexplored [20]. Increasing Hg concentrations could potentially impact the relative abundance of major ecological clusters with implications for ecosystem functioning. However, empirical evidence for such assumptions is currently lacking.

Moreover, the response of the functional attributes of the soil microbiome to Hg pollution has been rarely addressed. Such a task is challenging as soil microbial communities are highly diverse, and most soil microbial taxa remains uncharacterized [16, 21]. Recent advances in metagenomic sequencing can infer the functional traits of microbial communities [22, 23] and then determine which are sensitive to increased soil Hg pollution. We expect functional traits of soil microbial communities to follow predictable responses to Hg pollution. Such an expectation is based on the large body of literature reporting consistent microbial responses to other global changes such as climate change and nutrient additions [3, 23, 24].

Herein, we aimed to build a predictive understanding of the response of multiple soil taxonomic and functional attributes to increased Hg contents at the regional scale. Because Hg is a well-known pollutant with important implications for life on Earth [25], we hypothesized that soil Hg pollution will have consistent effects on taxonomic and functional attributes (e.g., relative abundance of functional genes) across different land use types. To do so, we collected three replicated soils at each of 47 sites (24 paddy fields and 23 adjacent upland fields), resulting in a total of 141 samples from Hg-impacted agricultural locations across two provinces in China. Most of the sites are surrounded by historical Hg mining areas with varying soil Hg contents, and their soils contained a wide range of Hg concentrations under similar vegetation types. These sites also included controls that are far away from the mining sites, with Hg contents similar to local background levels. As such, these locations provided a unique opportunity to empirically evaluate the response of microbial taxonomic and functional attributes to a gradient of soil Hg pollution. We characterized soil bacterial community composition via MiSeq Illumina platform. In addition, we investigated potential shifts in the relative abundance of functional genes linked to microbial communities by analyzing the soil metagenomes from a subset of those sites. Finally, we identified the associations between soil Hg concentrations and bacterial diversity, abundance, and ecological clusters (modules) using a combination of random forest analysis and structure equation modeling, after accounting for other critical environmental predictors.

## Results

### Mercury pollution altered soil bacterial abundance and diversity

**Fig. 1:**
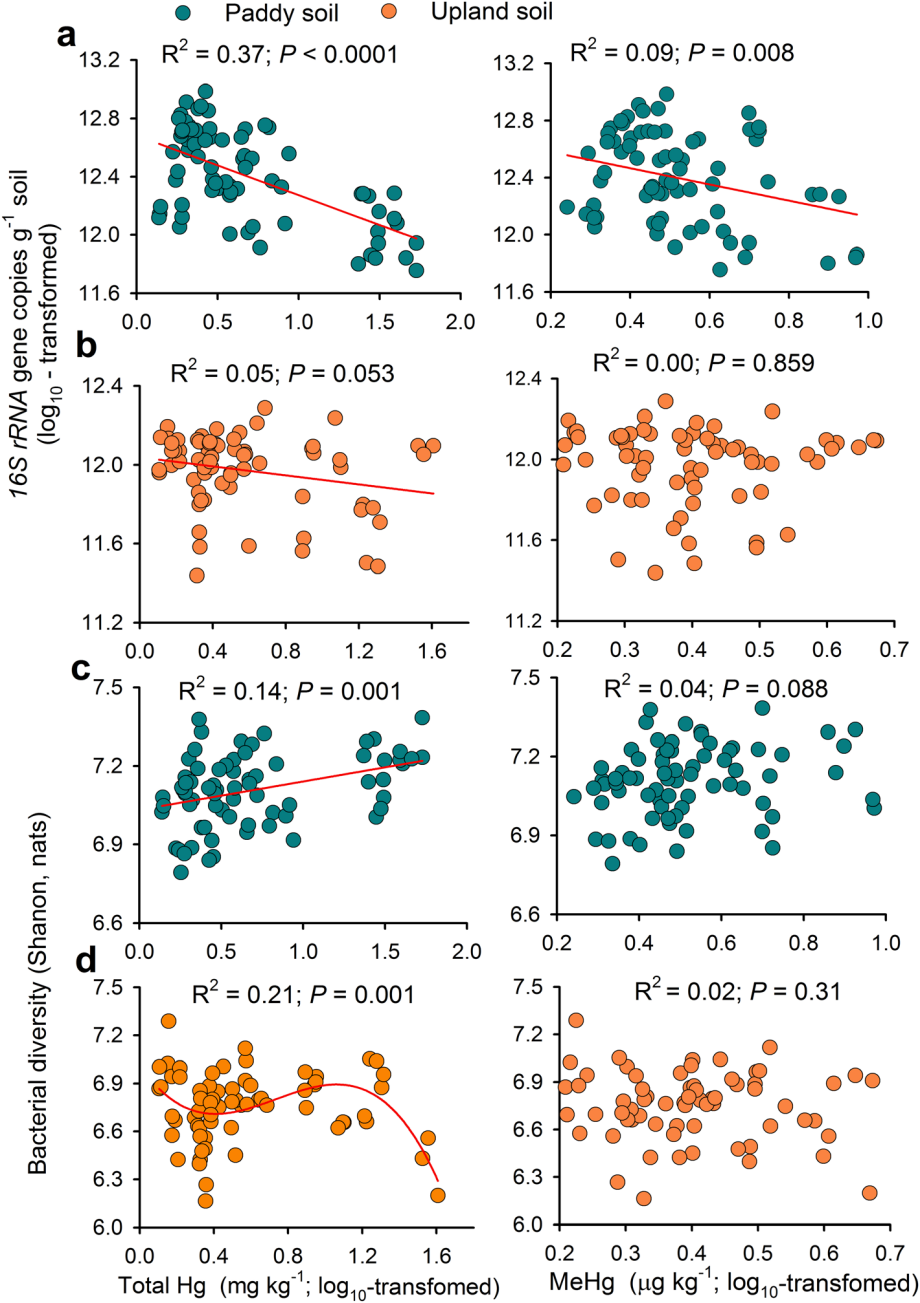
Relationships between soil mercury (Hg, including total Hg and methylmercury, MeHg) and the bacterial abundance (**a**, **b**), and diversity (**c**, **d**) in paddy and upland soils. Green and yellow dots represent samples from paddy and upland fields, respectively. Red lines represent regressions with linear (straight) and cubic (curve) functions (*P* < 0.05)

We found a consistent decrease in the abundance of bacteria with increases in soil total Hg in both paddy and upland soils (Fig. 1a, b). Bacterial abundance was also negatively related to methylmercury (MeHg) in paddy soils (d.f. = 1, 70, *P* = 0.008). In contrast, bacterial diversity (Shannon index) was positively correlated to total Hg (d.f. = 1, 70, *P* = 0.001) in the paddy soils and tended to increase with elevated MeHg contents (d.f. = 1, 70, *P* = 0.08) (Fig. 1c, d). We also found a cubic negative relationship (d.f. = 3, 65, *P* = 0.001) between total Hg and bacterial diversity for the upland soils. As expected, land use type had significant effects on soil bacterial abundance and diversity (Additional file 1: Figure S1a, b), and both bacterial abundance and diversity were higher (ANOVA, d.f. = 1, 139, *P* < 0.05) in paddy soils than those in upland soils.

### Mercury pollution altered the relative abundance of bacterial phyla

**Fig. 2:**
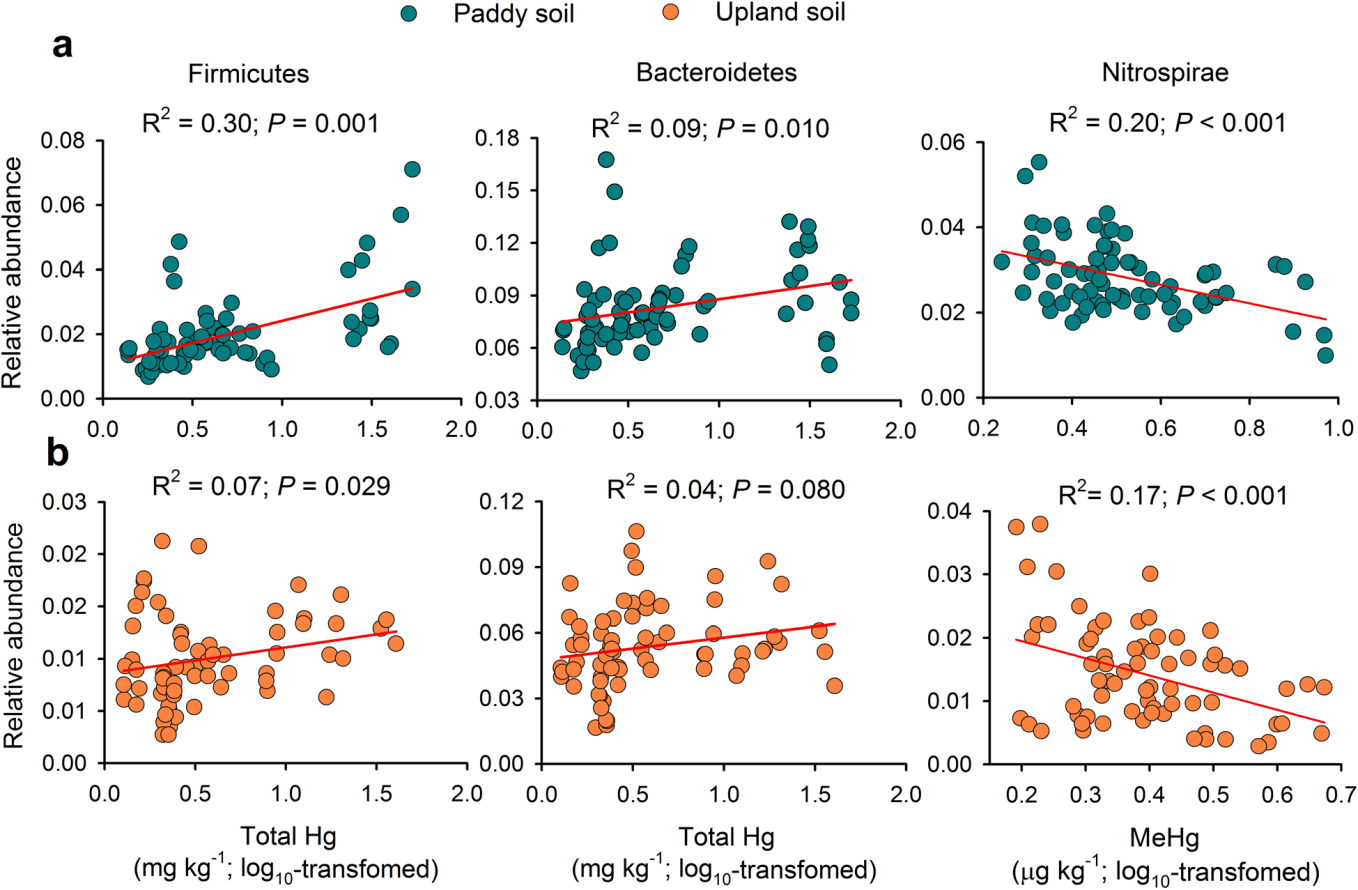
Linear relationships between soil Hg contents and the relative abundance of selected bacterial phyla in paddy (**a**) and upland (**b**) soils. Correlations between Hg concentrations and the relative abundance of all major bacterial phyla are available in Additional file 1: Table S1

We found consistent associations between of Hg contents and the relative abundance of some dominant bacterial phyla in both paddy and upland fields, though the bacterial community compositions were different between the two types of land use (Additional file 1: Figure S2). For example, increasing total Hg was positively related to the relative abundance of *Firmicutes* and *Bacteroidetes* in both paddy and upland soils (Fig. 2), while they were not related to MeHg. In contrast, increasing MeHg was negatively correlated to the relative abundance of *Nitrospirae* in the two soils, though it was not significantly correlated to soil total Hg (*P* > 0.05). In addition, we found some correlations, depending on land use type, between total Hg/MeHg and the relative abundance of other dominant phyla/classes (Additional file 1: Table S1). Random forest analysis allowed us to further identify what genera of *Firmicutes* and *Bacteroidetes* were associated with increases in Hg contents (Additional file 1: Figure S3a, *P* < 0.05). Thus, the relative abundance of *Chitinophagaceae Ferruginibacter*, *Sphingobacteriaceae Pedobacter*, and *Clostridium* sensu *stricto*_1 were positively correlated to soil total Hg (Additional file 1: Figure S3b, d.f. = 1, 139, *P* < 0.05), while the relative abundance of *Nitrospirae* was negatively correlated to MeHg contents (Additional file 1: Figure S4, d.f. = 1, 139, *P* < 0.01).

### Mercury pollution shifted the relative abundance of ecological clusters within the correlation network

**Fig. 3:**
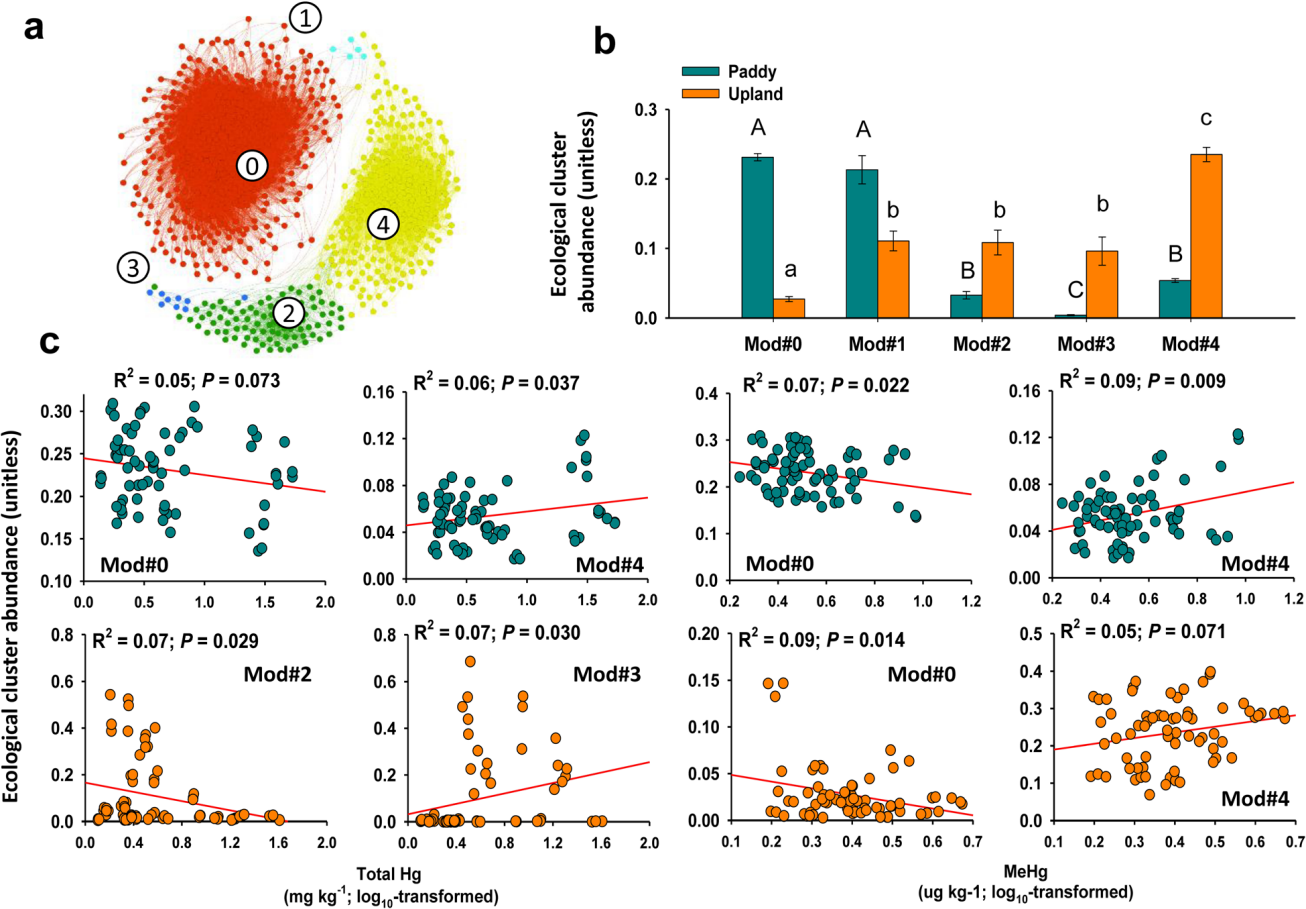
Microbial correlation network. Panel **a** represents a network diagram with nodes (taxa) colored by each of the major five ecological clusters (modules, Mod) within co-occurrence network of bacterial communities; Panel **b** includes the relative abundance of the five modules. Capital letters indicate the significant differences between the paddy soils (*P* < 0.05). Lowercase letters indicate the significant differences between the upland soils (*P* < 0.05); Panel **c** includes the linear relationships between soil Hg pollution (total Hg and MeHg) and the relative abundance of the selected ecological clusters

The soil bacterial taxa could be grouped into five major ecological clusters (modules), comprised of strongly co-occurring bacterial taxa with one another (Fig. 3a). Not surprisingly, the two different soils were dominated by different ecological clusters in the two types of soil (Fig. 3b). Particularly, the relative abundance of module #0 was much higher (ANOVA, d.f. = 1, 139, *P* < 0.05) in the paddy soils than that in the upland soils, while the relative abundance of module #4 exhibited the opposite trend. Even so, we found consistent relationships between soil total Hg/MeHg and the relative abundance of the dominant ecological clusters (Fig. 3c). For example, the relative abundance of module #0 was negatively related to MeHg in both of soils, where the relative abundance of module #4 was positively related to MeHg. All modules were formed by multiple phyla/classes, and the membership of each module is shown in Additional file 2 (data S1). We also found some soil-type-specific effects of Hg on the relative abundance of ecological clusters. For instance, the relative abundance of modules #0 and #4 decreased and increased, respectively, with increasing total Hg in the paddy soil (Fig. 3c), but no similar trends were observed in the upland soil. Meanwhile, the relative abundance of module #2 and module #3 decreased and increased, respectively, with increasing total Hg for the upland soil, while no similar response was observed for the paddy soil.

### Mercury pollution is a significant predictor of soil bacterial community and ecological clusters after controlling for multiple environmental predictors

Random forest analysis suggested that, in general, Hg variables are major predictors of bacterial diversity and abundance (Additional file 1: Figure S5a, b) and the relative abundance of ecological clusters (Additional file 1: Figure S6a, b). As expected, our results indicate that other environmental factors were also important predictors of microbial communities, although the relative importance of these predictors was highly taxa and module dependent. See Additional file 1: Table S2 for correlations between microbial attributes and environmental predictors.

**Fig. 4:**
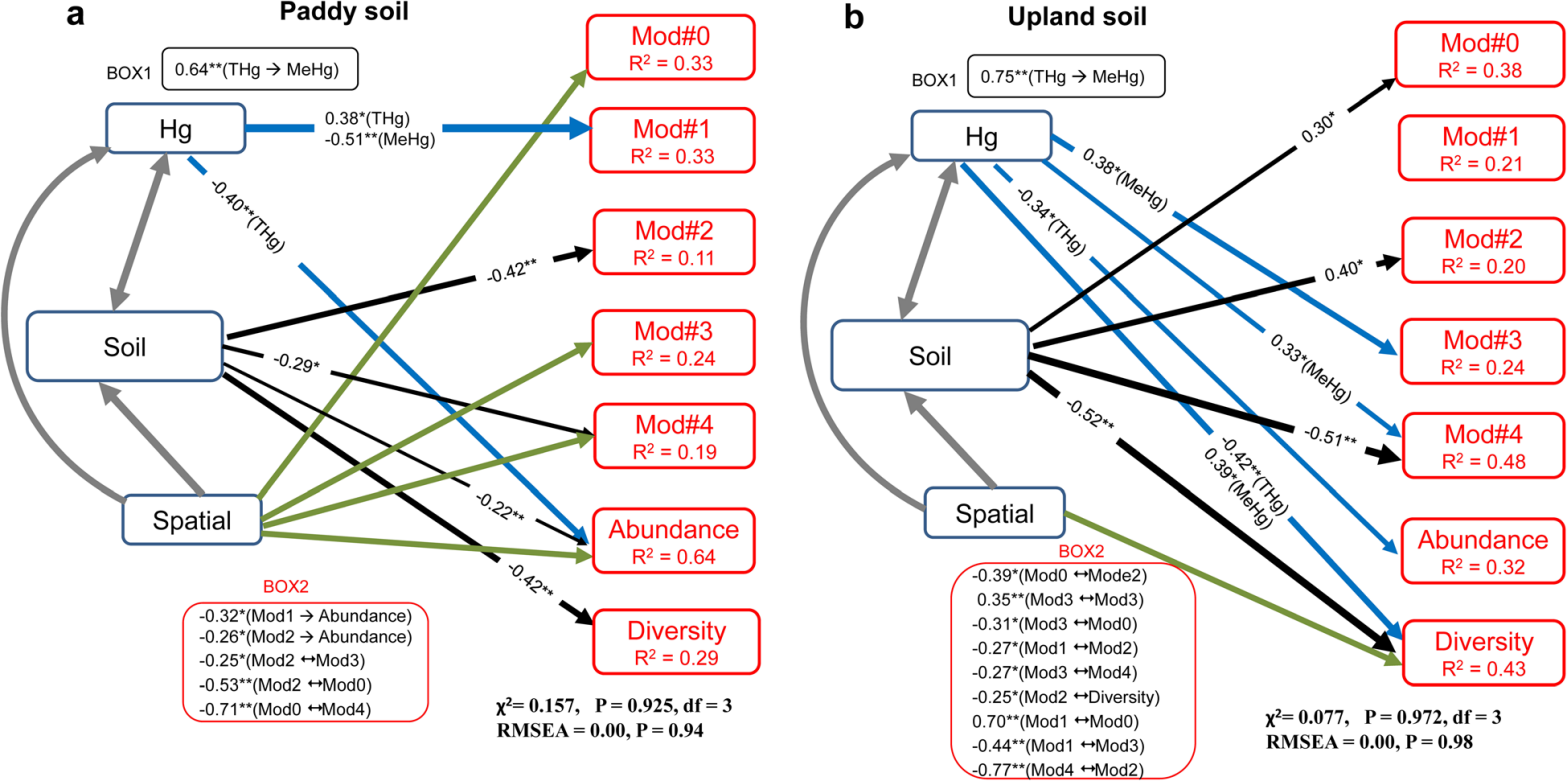
Mechanistic modeling identifying the direct and indirect effects of Hg on bacterial abundance, diversity, and the relative abundance of ecological clusters (modules, Mod) within co-occurrence networks in paddy (**a**) and upland (**b**) soils. The Hg box includes total Hg and methylmercury, and the spatial box includes longitude and latitude. The soil box includes soil properties that were represented by the three major components by performing principal component analysis of soil variables including pH, soil organic carbon (SOC), C: N, and others (Additional file 1: Table S3). The thickness of the arrow represents the strength of the relationship when significant, while no arrow is showed when the effect is not significant. Numbers adjacent to arrows are path coefficients with significant levels. *R*^2^ denotes the proportion of variance explained. Spatial (latitude and longitude) influence was included to control spatial autocorrelation; however, in this case, path coefficients were not included for simplicity. The BOX2 includes the significant correlations between modules, diversity, and abundance. The rest of significant effects are available in Additional file 1: Table S4 (*P* < 0.05).*P < 0.05, **P < 0.01

We then used structural equation modeling (SEM), to further clarify the role of Hg in predicting abundance, diversity, and the relative abundance of modules for both paddy and upland soils, after controlling for multiple other environmental predictors (Fig. 4a, b; Additional file 1: Table S3, S4). Remarkably, we found a consistent negative effect of total Hg on bacterial abundance in both soils. Our SEM shows direct effects of MeHg on the relative abundance of modules (Mod#3 and Mod#4) in upland soils and effects of total Hg and MeHg on the relative abundance of Mod#1 in paddy soils. Interestingly, we detected multiple indirect effects of Hg on the relative abundance of these modules via impacts on soil properties and other modules, though there may be interactions between these microbial attributes. The SEM also shows a direct effect of Hg on the bacterial diversity in upland soils, while the effect was not significant in the paddy soils. As expected, the SEM also shows strong effects of soil and spatial properties on these microbial attributes across the two types of land use.

### Functional gene relates to soil Hg pollution

**Fig. 5:**
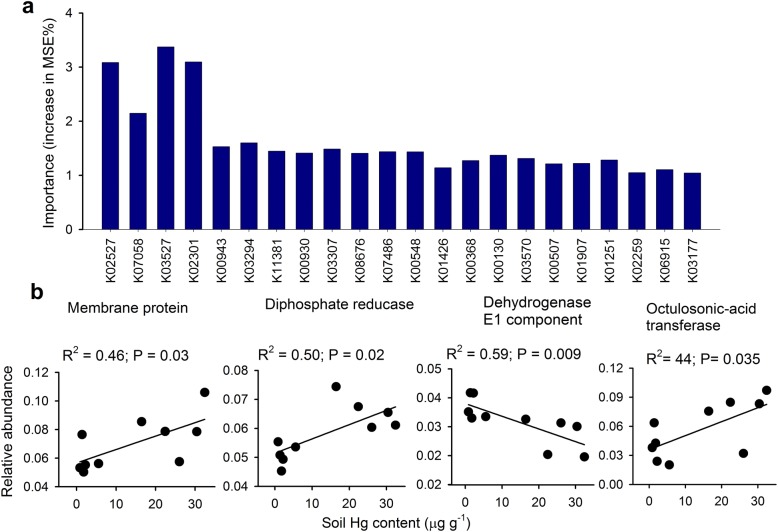
Random forest (RF) analyses identifying the significant (*P* < 0.05) gene predictors of soil total Hg (**a**). Panel **b** includes relationships between total Hg contents and selected functional genes. These functional genes were annotated according to Kyoto Encyclopedia of Genes and Genomes (KEGG) using metagenomic data derived from a subset of our soil samples. Additional information on the KEGG genes is available in Additional file 1: Table S5

We conducted further random forest modeling to evaluate the link between functional genes and Hg pollution, and 22 functional genes that significantly predicted changes in total Hg across the soils were identified (Fig. 5a; Additional file 1: Table S5). Most of those genes are likely involved in soil nutrient metabolisms (e.g., reduction of nitrate and phosphate) and Hg transformations (e.g., Hg uptake and methylation). Furthermore, we found that the relative abundance of genes encoding member protein, diphosphate reductase, and dehydrogenase E1 component increased along elevated soil Hg contents, while the gene encoding 3-deoxy-d-manno-octulosonic-acid transferase accounting for glycan biosynthesis and metabolism, significantly decreased towards increased Hg (Fig. 5b). We also found 30 genes significantly predicting changes in soil MeHg, including the genes involved in CoA synthetase and iron transport, that are important enzymes involved in Hg uptake and methylation (Additional file 1: Figure S7).

## Discussion

### Effects of Hg pollution on soil bacterial abundance and diversity in agricultural ecosystems

Both regression analysis and SEM demonstrate that long-term field Hg pollution had a negative effect on the bacterial abundance from the two land use types. Such a result is in contrast with a previous short-term incubation experiment where no effect of Hg pollution on bacterial abundance was found [13]. Both Hg and MeHg are toxic to life, and thus, long-term field Hg pollution may inhibit microbial growth and then cause a decrease in soil microbial abundance across centuries [8]. Importantly, the lack of an effect of Hg pollution on bacterial abundance in short-term experiments could be related to the fact that relic DNA from dead bacteria is also extracted and then quantified by qPCR [26, 27], obscuring the adverse effect of Hg on soil microbial abundance. Such an artifact should be largely diluted in long-term experiment and observational studies. Similarly, MeHg as the most toxic Hg speciation also negatively correlated to the bacterial abundance in the paddy soils, further emphasizing the strong effect of Hg pollution on bacterial abundance in soil. On the contrary, bacterial diversity tended to increase under moderate Hg content (< 53 mg kg^−1^, Fig. 1c), partially supporting the intermediate disturbance or stress hypothesis [12, 28]. However, our observations are different from those from short-term incubation experiments, in which Hg amendment has been reported to reduce soil bacterial diversity [13]. As such, our approach suggests that large-scale observational studies are needed to understand the response of bacterial diversity to Hg pollution in real-world ecosystems. The soils examined in this study belong to Hg-mining areas which suffered serious Hg pollution for more than 600 years [29]. Consequently, the microbial community from these soils might have had time to develop resistance to Hg stress [13]. Alternatively, increasing Hg pollution might increase bacterial diversity by releasing subordinate microbial taxa via competition. These results are partially supported by our SEM suggesting a positive effect of MeHg on the diversity in upland soils, while the effect became complicated in paddy soils where no direct effect of total Hg or MeHg on the diversity was observed. However, Hg pollution may indirectly influence the bacterial diversity through impacts on soil properties, which were important drivers of soil microbial diversity [17, 21]. All of these observations illustrate the importance of Hg pollution in regulating the abundance and diversity of soil bacteria. Importantly, these results were maintained after accounting for multiple other drivers including soil and spatial properties.

### Mercury pollution altered soil bacterial community composition and the distribution of ecological clusters

Importantly, we also found that Hg pollution consistently related to the relative abundance of the dominant bacterial phyla. For example, the relative abundance of *Firmicutes* and *Bacteroidetes* increased in response to elevated total Hg, but the relative abundance of *Nitrospirae* was negatively related to MeHg contents. *Firmicutes* and *Bacteroidetes* are known to be fast-growing opportunistic types of organisms that might benefit from environmental disturbance by taking over niches commonly occupied by other bacterial taxa. We also detected the genera of bacteria within these groups influenced by Hg pollutions (Additional file 1: Figure S3; Figure S4). For example, *Sphingobacteriaceae Pedobacter* and *Clostridium* sensu stricto have been reported to be resistant to Hg or heavy metals [30, 31]; however, much less is known about the mechanisms linking the responses of *Chitinophagaceae Ferruginibacter* to Hg pollution. In addition, our large-scale data suggest a decrease in the relative abundance of *Nitrospirae* with elevated soil MeHg. Previous studies have found that this phylum is highly sensitive to heavy metal stress at the local scale [12, 32]. The reduction in the relative abundance of *Nitrospirae* was mainly attributed to a relative decrease in the genus *Nitrospirales* 0319.6A21sp. in response to elevated MeHg. Increased Hg pollution is known to inhibit the processes of nitrogen cycle driven by soil functional assembles [9, 33], which could be an important result of the inhibiting effect of Hg to nitrifying bacteria such as *Nitrospirae*. These results suggest that the sensitive taxa could be used as potential ecological indicators for Hg pollution in terrestrial ecosystems.

Our random forest analysis suggested that Hg, in general, is a significant predictor of the ecologically preferential modules within the bacterial co-occurrence network after accounting for other key environmental predictors (Additional file 1: Figure S6). Thus, increases in soil Hg content also led to drastic changes in the co-occurrence network of soil bacterial communities in the studied regions. Interestingly, we found significant patterns of the relative abundances in some modules along increased Hg and MeHg pollution (Fig. 3c; Additional file 1: Table S6). For example, the relative abundance of module #4 tended to increase with elevated total Hg and MeHg in both paddy and upland soils. This module mainly consisted of potentially Hg-resistant microbes such as *Proteobacteria*, *Bacteroidetes*, and *Actinobacteria*, which were reported to contain the Hg-resistance gene *merA* [34, 35]. We also provide a list of winner (positive effects) and loser (negative effects) community assemblies in response to Hg pollution (Additional file 2: Data S1), which can be used to test for similarities in the response of soil microbial communities to Hg pollution worldwide.

Although SEM is quite a conservative procedure, the results support similar associations between Hg and the relative abundance of modules across the two types of land use. For example, soil Hg had direct effects on the relative abundance of module #1 in paddy soils (Fig. 4a), and a similar effect on this module in upland soils could be reflected through indirect impacts of Hg on other modules (i.e., Mod#2, Mod#3, and Mod#4) due to their interactions (Fig. 4b: BOX2). It is not surprising to note the interactions between the modules in the co-occurrence networks, because increases in a given ecological cluster were often followed by declines in the relative abundance of other ecological clusters [36]. These findings suggest that variability of Hg contents in the ecosystem might change the distributions of ecologically preferential clusters. Of course, as expected, other environmental predictors strongly associate with the relative abundance of microbial ecological clusters (Fig. 4, Additional file 1: Table S2; Additional file 1: Figure S6), which had been well recognized in many previous studies [15–17].

### Predicting soil Hg pollution from the variability of microbial functional genes

We found that Hg pollution was also associated with important functional traits within microbial communities. In fact, here, we provide a list of functional genes which were strongly correlated to Hg contents across soils. Of course, we acknowledge the current limitations for linking genes to Hg pollution across large spatial scales due to the correlative nature of these analyses (Fig. 5; Additional file 1: Figure S7). Such results are in agreement with previous studies emphasizing significant effects of Hg stress on microbially driven processes (e.g., nitrification potential and dehydrogenase enzyme activity) driven by diverse soil microorganisms [9, 37]. For example, the relative abundance of genes involved in member protein tended to increase with the level of soil Hg pollution, suggesting that moderated Hg stress may stimulate the enzyme activities responsible for Hg transportation through the cell membrane [38]. We also observed a significant increase in the relative abundances of genes associated with dehydrogenase with elevated Hg. These findings are consistent with those of previous studies showing upregulation of dehydrogenase activities exposed to metals [39]. In addition, we identified some genes that are relevant to Hg transport (i.e., iron transport system) and methylation processes (i.e., CoA synthetase) [40]. Previous studies has suggested that inorganic Hg(II) could be transported into microbial cells probably through ion transport system [41, 42], and the cellular Hg(II) is subsequent methylated to highly neurotoxic MeHg by methylating genes *hgcAB* [43]. Thus, our predicted iron transport system might be relevant to soil MeHg formation. Furthermore, Hg methylation is an enzyme-catalyzed process associated with the reductive CoA pathway [44], which may explain our results that the CoA synthetase is a significant predictor for MeHg in the soil. Overall, these gene predictors are associated with nutrient metabolisms and Hg transformations, which are also important biomarkers of soil Hg pollution.

## Conclusions

Together, our study represents one of the first attempts to empirically assess how the soil microbiomes respond to long-term Hg pollution across large spatial scales and land use type. We provides solid evidence that Hg pollution has predictable and significant effects on multiple taxonomic and functional microbial attributes including bacterial abundance, diversity, and the relative abundance of ecological clusters and functional genes. Such results are maintained after accounting for other important environmental predictors of soil microbial communities. In general, Hg pollution was negatively related to the bacterial abundance. The relative abundance of *Bateroidetes* and *Firmicutes* increased with elevated Hg pollution, while the relative abundance of *Nitrospirae* was negatively correlated to MeHg. Mercury-induced shifts in the ecological clusters of co-occurrence network and functional traits of microbial communities could have important implications for soil biogeochemical cycling and service functioning of the ecosystem. This work moves us towards a more predictable understanding of the response of microbial communities and their potential function to Hg pollution—a worldwide threat derived from both global warming and intensive anthropogenic activities.

## Methods

### Study area and sampling

The soil samples were collected around two typical Hg mining areas (Fenghuang, FH and Wanshan, WS) in southwest China. Fenghuang is located in West Hunan Province, and Wanshan in East Guizhou Province. Both of them are the major grain-producing areas in China, with a long history Hg mining for more than 600 years [29]. Historical discharges from Hg mining operations and ongoing atmospheric deposition has led to heavy Hg pollution in these areas [45]. We collected soil samples from 24 locations (Additional file 1: Figure S8) along downstream the mining sites to obtain samples with a large gradient of soil Hg concentrations (ranges from 0.27 to 52.4 mg kg^−1^), and most of them were above risk control value for paddy soils in China (0.6 mg kg^−1^) [46]. Two typical types of land use including paddy fields (24 sub-sites) and adjacent uplands (23 sub-sites, maize planting) were chosen to compare the effects of land use on the microbial responses to long-term soil Hg pollution. We tried to choose locations where both paddy and upland fields are the main crop planting, and sporadic farmlands are excluded. Three replicated soil (0–15 cm depth) samples were collected at each of the 47 sites to account for small scale variation in Hg pollution. Consequently, a total of 141 soil samples were obtained from the 24 paddy (72 samples) and upland (69 samples) fields. These locations also included two control sites (paddy and adjacent upland fields) located in the natural reserve with little pollution (i.e., location no. 1 in Additional file 1: Figure S8). Total Hg concentrations in the control sites are similar the levels of local background values (0.5~0.7 mg kg^−1^), which are similar to the Hg risk control value for paddy soil in China [46]. We omitted an upland site due to the lack of representative field in the location. Sampling was conducted in August 2016 before harvesting. Collected soil samples were homogenized and sieved (2.0 mm) to get a representative microbial community [20, 47]. The sieved samples were subsequently divided into two sub-samples. One sub-sample was stored at − 20 °C for microbial analysis, while the other sub-sample was air-dried for the analysis of heavy metals and soil properties.

### Measurement of soil heavy metals and chemical indexes

For Hg analysis, 0.20 g of each soil was digested with 10 mL mixed solution (2 mol L^−1^ HNO_3_ and 4 mol L^−1^ HCl) in a Teflon tube at 95 °C for 2 h. The total amount of Hg in these extracts was determined via cold vapor atomic fluorescence spectrometry (CVAFS) [45], and the method detection limit was 0.2 ng L^−1^. For the analysis of other heavy metals (i.e., Cu, Pb, Cd, Zn, Ni, and As), 0.30 g soil were digested by trace mixed acids (9.0 mL of HNO_3_ and 3.0 mL of HF) in a MARS microwave digestion system (CEM, USA). The concentrations of the heavy metals in the final solution were measured using a 7700X Inductively Coupled Plasma-Mass Spectrometer (Agilent, USA). For MeHg, 0.40 g soil was used for MeHg extraction using CuSO_4_-methanol. The amount MeHg was determined using an automated MeHg analyzer (TEKRAN 2700 GC-CVAFS) [48]. Soil pH was determined on a fresh soil to water ratio of 1: 2.5 using a Delta pH-meter. Soil organic carbon (SOC) was measured using the K_2_CrO_7_ oxidation titration method [49]. Total carbon (TC) and total nitrogen (TN) in soils were determined on a LECO TureMac Macro CN analyzer (LECO, St. Joseph, MI, USA). Dissolved organic carbon (DOC) in the soil was extracted with 0.5 M K_2_SO_4_ at a ratio of 1:5 by shaking at 200 rpm for 1 h and filtering through a 0.45-μm Millipore filter [50], and the DOC concentration was measured by a TOC analyzer (TOC-L Analyzer, Shimadzu, Japan). The ammonium and nitrate concentrations in the filtered extracts were analyzed within 24 h using a continuous flow analyzer (SAN++, Skalar, Breda, Holland).

### Analysis of soil bacterial communities through Illumina MiSeq sequencing

The genomic DNA was isolated from 0.30 g of soil using the MoBio PowerSoil DNA Isolation Kit (QIAGEN Inc., USA) following the manufacturer’s instructions. The abundance of bacteria was estimated by quantifying the *16S rRNA* gene copy number on an iCycler iQ5 thermocycler (Bio-Rad, USA) using the primer pairs Eub338F/Eub518R [51]. To evaluate the bacterial community composition, the V4 region of the bacterial *16S rRNA* gene was amplified using the primer pairs of 338F/806R [47]. The 50 μl PCR reaction mixtures consisted of 25 μl PremixTaq™ (Takara Biotechnology, Dalian, China), 1 μl of each primer (10 μM), 3 μl of template DNA, and 20 μl of sterilized ddH_2_O. The resultant PCR products were purified using the Wizard® SV Gel and PCR Clean-Up System (Promega, San Luis Obispo, USA). The purified amplicons were equimolarly mixed, and 2 × 250 bp paired-end sequencing was carried out on an Illumina Miseq sequencer (Illumina Inc., San Diego, USA). Raw reads generated from the MiSeq paired-end sequencing were merged together using the Fast Length Adjustment of Short reads (FLASH). A chimera filtering approach UPARSE was employed as the Operational Taxonomic Unit (OTU or phylotype) picking strategy at 97% sequence similarity. We randomly selected an even number (30,212) of reads from each sample to account for variability in sequencing depth before downstream analysis (Additional file 1: Figure S9). Representative sequences from individual OTUs generated in UPARSE were processed using the Quantitative Insights into Microbial Ecology (QIIME) pipeline. The bacterial diversity index was calculated based on 97% OTU similarity of obtained bacterial sequences. The taxonomic identity of all phylotypes was determined using The SILVA ribosomal RNA gene database project [52].

### Shotgun metagenomic sequencing and gene analysis

Seven paddy soils and three upland soils were selected from our 141 samples for metagenomic analysis. These samples were chosen, after Hg analysis, to produce a wide range of Hg concentrations (ranged from 0.84 to 32.43 mg kg^−1^). Sequencing was performed using an Illumina PE150 (Illumina Inc.) at Majorbio, Inc., Shanghai, China. Raw reads (150 bp in length) were trimmed to remove low-quality reads. Paired reads of shotgun metagenomic sequences were merged with FLASH using default parameters [53]. Using MBLASTX, merged reads were also mapped against the protein sequence of the KEGG database (*E* value cutoff 1e^−6^), and the relative abundance of each KO gene was also calculated. Additional details on methodology are provided in the Additional file 3. To estimate the influence of elevated Hg and MeHg contents on these genes, we focused on the KO genes related to microbial metabolism, Hg transformations, and other sensitive ones.

### Correlation network analyses

We established a co-occurrence network to identify ecological clusters of bacterial taxa across the collected 141 soil samples. A single correlation network including all samples was conducted so that the identified ecological clusters are directly comparable across land use types. We kept those taxa accounting for more than 80% of the relative abundance of soil bacteria (1073 bacterial taxa in Additional file 2: Data S1). We then calculated all pairwise Spearman’s rank correlations (*ρ*) between all soil bacterial taxa using the psych package of the R statistical software (http://cran.r-project.org/) and focused exclusively on positive correlations as they provide information on bacterial taxa that may respond similarly to environmental conditions [54]. We considered a co-occurrence to be robust if the Spearman’s correlation coefficient was > 0.25 and *P* < 0.01 [17]. The network was visualized with the interactive platform Gephi [55]. Finally, we used default parameters from the interactive platform Gephi to identify ecological clusters (i.e., modules) of soil taxa strongly interacting with each other [55]. The relative abundance of each ecological cluster was computed by averaging the standardized relative abundances (z-score) of the taxa that belong to each ecological cluster [17].

### Statistical analysis

We first identified the correlations between total Hg and MeHg with soil bacterial abundance, diversity (Shannon), and the relative abundance of ecological clusters in both paddy and upland soil, using linear or cubic models. We used both regression analyses and Pearson correlation to evaluate correlations between total Hg and MeHg and the relative abundance of ecological clusters, and bacterial community composition for two land use types using one ANOVA, with land use type as the fixed factor. We conducted a classification random forest analysis [56, 57] to identify the statistically significant predictors of the bacterial diversity, total abundance, and the relative abundance of ecological clusters. The major aim of these analyses were to test whether total Hg or MeHg pollution are significant predictors of microbial attributes, after accounting for other key environmental predictors including location, soil properties, heavy metals, and nutrient availability. We also used random forest analysis to identify the genus predictors of the phyla/classes that were significantly correlated to soil Hg. The random forest model determined the importance of each predictor variable via evaluating the decrease in prediction accuracy (i.e., increase in the mean square error between observations and OOB predictions) when the data for that predictor are randomly permuted, as previously described [58]. These analyses were conducted using the rfPermute package [59] of the R statistical software (http://cran.r-project.org/). Additionally, we used Spearman’s correlation analyses to further evaluate the correlations between bacterial diversity (Shannon), total abundance, and the relative abundance of ecological clusters with environmental predictors. We also used random forest analysis to identify the major functional genes predicting the concentrations of Hg and MeHg in our soils. In these analysis, functional genes are used as predictors of Hg concentrations. After this, we used linear regressions to evaluate the direction of the relationships between the relative abundance of selected genes and soil total Hg concentrations.

We then used structural equation modeling (SEM) [60] to further clarify associations of Hg (total Hg and MeHg) contents with the bacterial abundance, diversity, and the relative abundance of ecological clusters (modules). Unlike the analysis of regression or ANOVA, SEM allows the ability to separate multiple effect pathways and consider them as parts of a system and thus is useful for isolating the complex relationships among environmental factors commonly found in natural ecosystems [60, 61]. The probability that a path coefficient differs from zero was tested using bootstrap resampling. Bootstrapping is preferred to the classical maximum-likelihood estimation in these cases because in bootstrapping, probability assessments are not based on the assumption that the data match a particular theoretical distribution. Our model also includes spatial autocorrelation (latitude and longitude) and soil properties, which were represented by the three major components by performing principal component analysis of soil variables including pH, soil organic carbon (SOC), C: N, and others (Additional file 1: Table S3). We first established an a priori model according to our current knowledge of environmental variable impacts on soil microbiomes (Additional file 1: Figure S10). The data matrix was fitted to the model using the maximum-likelihood estimation method. There is no single universally accepted test of overall goodness of fit for SEM. Thus, we used the chi-square test (χ^2^; the model has a good fit when 0 ≤ χ^2^/d.o.f ≤ 2 and 0.05 < *P* ≤ 1.00) and the root mean square error of approximation (RMSEA; the model has a good fit when RMSEA 0 ≤ RMSEA ≤ 0.05 and 0.10 < *P* ≤ 1.00 [62]. The SEM analyses were performed using AMOS 21.0 (SPSS Inc., Chicago, IL, USA).

## Additional files

Additional file 1: **Table S1.** Correlation coefficients (Pearson’s *ρ*) between the dominant phyla/classes and soil Hg and MeHg contents. **Table S2.** Correlation coefficients (Spearman’s *ρ*) between bacterial abundance, richness and modules, and soil properties. **Table S3.** Correlation coefficients (Spearman’s *ρ*) between three major components from principal component analysis and soil properties. **Table S4.** Standardized direct effects from the SEM in Fig. 4. **Table S5.** The ID and names of the predicted functional genes (see Fig. 4) were annotated according to Kyoto Encyclopedia of Genes and Genomes (KEGG) using metagenomic data derived from a subset of our soil samples. **Table S6.** Correlation coefficients (Pearson’s *ρ*) between bacterial modules (Mod) of co-occurrence network and soil Hg and MeHg contents. **Figure S1.** Bacterial abundance and diversity in paddy and upland soils from Hg-impacted FH and WS areas in China **Figure S2.** Relative abundances of the dominant phyla (classes) based on MiSeq sequencing of 16S rRNA gene in paddy and upland soils. **Figure S3.** Predictor importance of main genera in *Firmicutes* and *Bacteroidetes*es ponding to soil total Hg based on random forest analyses (a). Relationships between the predicted main species and soil total Hg (b). **Figure S4.** Relationship between the most important genus in *Nitrospirare* and soil MeHg. **Figure S5.** Random forest (RF) analyses identifying environmental predictors of soil bacterial abundance and diversity. **Figure S6.** Random forest analyses identifying soil environmental predictors of the relative abundance of modules #0–4 of bacterial occurrence network in paddy soils. **Figure S7.** Random forest analyses identifying the main significant gene predictors of soil total MeHg. **Figure S8.** Map of the study area and 24 sampling locations around the Hg mining area in southwest China. **Figure S9.** Rarefaction and Shannon curves of *16S rRNA* gene sequencing of the soils. **Figure S10.** An a priori model identifying effects of Hg on soil microbiomes. (DOCX 1450 kb)

Additional file 2: Taxa composition of each module (ecological cluster). (XLSX 48 kb)

Additional file 3: Details regarding shotgun metagenomic sequencing and gene analysis. (DOCX 18 kb)
