# Supplementary material for: Consistent responses of soil microbial taxonomic and functional attributes to mercury pollution across China
Source: Microbiome. 2018 Oct 18;6:183. doi: 10.1186/s40168-018-0572-7 (PMC6194565; doi:10.1186/s40168-018-0572-7)

**Table S1.** Correlation coefficients (Pearson’s ρ) between the relative abundances of dominant bacterial phyla/classes and soil total Hg and methylmercury (MeHg) contents. *P* values below 0.05 are in bold.

|  | Paddy soil (n = 72) | | | Upland soil (n = 69) | |  |
| --- | --- | --- | --- | --- | --- | --- |
|  | Total Hg | MeHg |  | Total Hg | MeHg | |
| *Alphaproteobacteria* | 0.07 | -0.03 |  | 0.01 | **0.35** | |
| *Betaproteobacteria* | **-0.24** | -0.08 |  | 0.19 | -0.01 | |
| *Deltaproteobacteria* | **-0.25** | **-0.27** |  | -0.06 | -0.06 | |
| *Gammaproteobacteria* | -0.03 | .006 |  | 0.15 | **0.24** | |
| *Actinobacteria* | **0.39** | **0.31** |  | 0.00 | 0.10 | |
| *Acidobacteria* | **-0.25** | -0.17 |  | -0.13 | **-0.35** | |
| *Chloroflexi* | -0.12 | -0.19 |  | **-0.28** | **-0.27** | |
| *Bacteroidetes* | **0.30** | **0.45** |  | 0.21 | 0.15 | |
| *Gemmatimonadetes* | 0.15 | 0.20 |  | **-0.31** | -0.08 | |
| *Nitrospirae* | **-0.28** | **-0.45** |  | -0.06 | **-0.39** | |
| *Firmicutes* | **0.55** | **0.47** |  | **0.26** | -0.07 | |
| *Verrucomicrobia* | 0.10 | 0.12 |  | -0.02 | -0.16 | |

**Table S2.** Correlation coefficients (Spearman’s ρ) between bacterial abundance, richness and modules (Mod) and soil properties. TC and TN, total carbon and total nitrogen, respectively; SOC, soil organic carbon; DOC, dissolved organic carbon; *P* values below 0.05 are in bold.

|  | **Abundance** | **Richness** | **Mod#0** | **Mod#1** | **Mod#2** | **Mod#3** | **Mod#4** |
| --- | --- | --- | --- | --- | --- | --- | --- |
|  | **Paddy soil** | | | | | | |
| Longitude | **-0.67** | 0.05 | 0.06 | **0.49** | **-0.32** | -0.22 | -0.15 |
| Latitude | **-0.43** | -0.09 | -0.05 | **0.44** | **-0.34** | **-0.30** | -0.13 |
| pH | -0.11 | **0.29** | **0.51** | -0.07 | **-0.35** | 0.2 | -0.04 |
| TC | **-0.43** | 0.01 | -0.13 | 0.21 | -0.15 | -0.14 | 0.06 |
| TN | **-0.44** | 0.06 | -0.15 | 0.2 | -0.11 | -0.17 | 0.04 |
| C:N | **-0.49** | 0.06 | -0.13 | **0.34** | -0.2 | -0.12 | 0.09 |
| SOC | **0.64** | **-0.249** | 0.380 | **-0.28** | **-0.30** | -0.05 | **-0.24** |
| NH_4_^+^ | 0.22 | -0.08 | -0.13 | **-0.35** | **0.31** | 0.1 | 0.03 |
| NO_3_^-^ | **-0.33** | -0.21 | **0.25** | **0.25** | **-0.46** | 0 | **-0.24** |
| DOC | 0.04 | -0.069 | **0.317** | **-0.41** | -0.136 | **0.29** | -0.125 |
| Ni | **0.38** | **-0.35** | 0.13 | **-0.28** | 0.01 | -0.03 | -0.12 |
| Cu | **0.26** | **-0.35** | **0.26** | -0.12 | -0.08 | -0.02 | **-0.29** |
| Cd | **-0.33** | -0.01 | **0.28** | 0.23 | **-0.28** | -0.03 | **-0.26** |
| Pb | **0.29** | -0.16 | 0.04 | -0.03 | -0.01 | -0.07 | -0.02 |
| As | **0.35** | **-0.32** | 0.02 | -0.2 | -0.01 | -0.07 | -0.03 |
| Zn | 0.04 | 0 | -0.04 | 0.2 | -0.12 | -0.13 | 0.13 |
|  | **Upland soil** | | | | | | |
| Longitude | 0.205 | **0.42** | **0.55** | **0.34** | 0.04 | **0.53** | **-0.45** |
| Latitude | **0.38** | **0.31** | **0.50** | **0.26** | 0 | **0.38** | **-0.30** |
| pH | 0.1 | -0.07 | 0.12 | 0.16 | **-0.75** | -0.03 | 0.51 |
| TC | -0.22 | -0.08 | -0.22 | -0.02 | **-0.29** | **-0.33** | 0.21 |
| TN | -0.14 | -0.1 | **-0.28** | -0.07 | **0.27** | **-0.44** | -0.02 |
| CN | -0.15 | -0.06 | -0.1 | 0.07 | **-0.38** | **-0.26** | **0.25** |
| SOC | -0.08 | 0.07 | **0.27** | **0.50** | -0.12 | **-0.47** | 0 |
| NH_4_^+^ | 0.11 | **0.36** | **0.41** | **0.28** | 0.04 | **0.47** | **-0.38** |
| NO_3_^-^ | -0.06 | **-0.38** | **-0.40** | -0.16 | -0.15 | **-0.59** | **0.47** |
| DOC | **0.41** | **-0.34** | **-0.42** | **-0.40** | -0.06 | -0.16 | **0.34** |
| Ni | 0.01 | -0.13 | 0.02 | 0.04 | -0.09 | -0.14 | 0.19 |
| Cu | -0.12 | 0 | 0.054 | 0.03 | -0.16 | 0.06 | 0.12 |
| Cd | -0.04 | 0.16 | 0.21 | **0.28** | -0.22 | 0.19 | 0.02 |
| Pb | -0.13 | -0.05 | 0.01 | 0.09 | -0.19 | -0.09 | 0.2 |
| As | 0.031 | -0.11 | **-0.26** | **-0.33** | 0.2 | -0.17 | 0.19 |
| Zn | 0.034 | **-0.25** | **-0.29** | -0.05 | **-0.35** | -0.11 | **0.44** |

**Table S3.** Correlation coefficients (Spearman’s ρ) between three major components extracted from Principal Component Analysis (PCA) and soil properties. TC and TN, total carbon and total nitrogen, respectively; SOC, soil organic carbon; DOC, dissolved organic carbon; *P* values below 0.05 are in bold.

| **Paddy soil** | | | |  | **Upland soil** | | |
| --- | --- | --- | --- | --- | --- | --- | --- |
|  | **PC1** | **PC 2** | **PC 3** |  | **PC1** | **PC 2** | **PC 3** |
| pH | 0.02 | -0.21 | **-0.33** |  | -0.12 | 0.12 | 0.11 |
| TC | 0.16 | **-0.27** | 0.17 |  | 0.00 | 0.13 | 0.04 |
| TS | 0.18 | **-0.24** | 0.22 |  | 0.11 | 0.13 | -0.03 |
| CN | 0.07 | **-0.39** | 0.15 |  | -0.10 | 0.20 | 0.03 |
| SOC | -0.06 | **0.39** | 0.16 |  | -0.04 | 0.02 | -0.08 |
| NH_4_^+^ | -0.15 | **0.25** | **-0.46** |  | -0.05 | -0.19 | 0.18 |
| NO_3_^-^ | -0.12 | **-0.24** | -0.20 |  | -0.16 | **0.34** | -0.17 |
| DOC | -.041 | 0.12 | **-0.93** |  | -0.19 | **0.99** | -0.07 |
| Ni | **-0.48** | **0.82** | 0.16 |  | **-0.48** | 0.22 | **-0.65** |
| Cu | **-0.52** | **0.75** | 0.14 |  | **-0.61** | 0.012 | **-0.67** |
| Cd | **-0.58** | 0.05 | 0.18 |  | **-0.63** | -0.13 | **-0.33** |
| Pb_ | **-0.80** | **0.55** | **0.26** |  | **-0.79** | -0.02 | **-0.45** |
| As | **-0.38** | **0.53** | 0.10 |  | **-0.34** | -0.05 | -0.03 |
| Zn | **-0.99** | 0.02 | 0.12 |  | **-0.96** | 0.15 | **0.24** |

**Table S4**. Standardized direct effects (*P* < 0.05) from the Structural Equation Modeling in Figure 4. PC means major components extracted from Principal Component Analysis (PCA) of soil properties (Table S3).

| **Response variables** | | **Predictors** | **Standardized effect** | **P-value** |
| --- | --- | --- | --- | --- |
| **Paddy soil** | | | | |
| PC2 | ← | Longitude | -0.796 | 0.01 |
| PC3 | ← | Latitude | 1.088 | <0.001 |
| PC3 | ← | Longitude | -0.794 | 0.013 |
| Mod#0 | ← | Latitude | -1.449 | <0.001 |
| Mod#3 | ← | Latitude | -0.758 | 0.027 |
| Mod#0 | ← | Longitude | 1.442 | <0.001 |
| Abundance | ← | Longitude | -0.743 | 0.002 |
| Mod#4 | ← | Longitude | -0.714 | 0.047 |
| Abundance | ← | PC1 | -0.219 | 0.007 |
| Diversity | ← | PC2 | -0.42 | <0.001 |
| Mod#4 | ← | PC2 | -0.293 | 0.029 |
| **Upland soil** | | | | |
| Diversity | ← | Longitude | 0.67 | 0.02 |
| Mod#0 | ← | PC1 | 0.296 | 0.021 |
| Mod#4 | ← | PC1 | -0.509 | <0.001 |
| Mod#2 | ← | PC1 | 0.401 | 0.006 |
| Diversity | ← | PC2 | -0.518 | <0.001 |
| Mod#4 | ← | PC2 | 0.257 | 0.015 |
| Abundance | ← | PC3 | 0.308 | 0.002 |
| Mod#4 | ← | PC3 | 0.224 | 0.011 |

**Table S5.** The ID and definition of the predicted functional genes (see Figure 5) were annotated according to Kyoto Encyclopedia of Genes and Genomes (KEGG) using metagenomic data derived from a subset of our soil samples.

| KEGG ID | KEGG gene |
| --- | --- |
| K02527 | 3-deoxy-D-manno-octulosonic-acid transferase |
| K07058 | membrane protein |
| K03527 | 4-hydroxy-3-methylbut-2-en-1-yl diphosphate reductase |
| K02301 | protoheme IX farnesyltransferase |
| K00943 | dTMP kinase |
| K03294 | basic amino acid/polyamine antiporter, APA family |
| K11381 | 2-oxoisovalerate dehydrogenase E1 component |
| K00930 | acetylglutamate/acetylaminoadipate kinase |
| K03307 | solute: Na+ symporter, SSS family |
| K08676 | tricorn protease |
| K07486 | transposase |
| K00548 | 5-methyltetrahydrofolate--homocysteine methyltransferase |
| K01426 | amidase |
| K00368 | nitrite reductase (NO-forming) |
| K00130 | betaine-aldehyde dehydrogenase |
| K03570 | rod shape-determining protein MreC |
| K00507 | stearoyl-CoA desaturase (delta-9 desaturase) |
| K01907 | acetoacetyl-CoA synthetase |
| K01251 | adenosylhomocysteinase |
| K02259 | cytochrome c oxidase subunit XV assembly protein |
| K06915 | uncharacterized protein |
| K03177 | tRNA pseudouridine synthase B |

**Table S6.** Correlation coefficients (Pearson’s ρ) between bacterial modules (Mod) within co-occurrence network and soil Hg contents. *P* values below 0.05 are in bold.

|  |  | Mod#0 | Mod#1 | Mod#2 | Mod#3 | Mod#4 |
| --- | --- | --- | --- | --- | --- | --- |
| Paddy soil | Total Hg | -0.21 | 0.071 | 0.04 | 0.08 | **0.25** |
|  | MeHg | **-0.27** | **-0.29** | 0.14 | 0.18 | **0.31** |
| Upland soil | Total Hg | -0.18 | -0.05 | **-0.26** | **0.26** | 0.13 |
|  | MeHg | **-0.29** | -0.14 | -0.12 | 0.18 | 0.22 |

**Figure S1.** Bacterial abundance (a) and diversity (b) in paddy (n = 72) and upland (n = 69) soils from Hg-impacted areas in China.


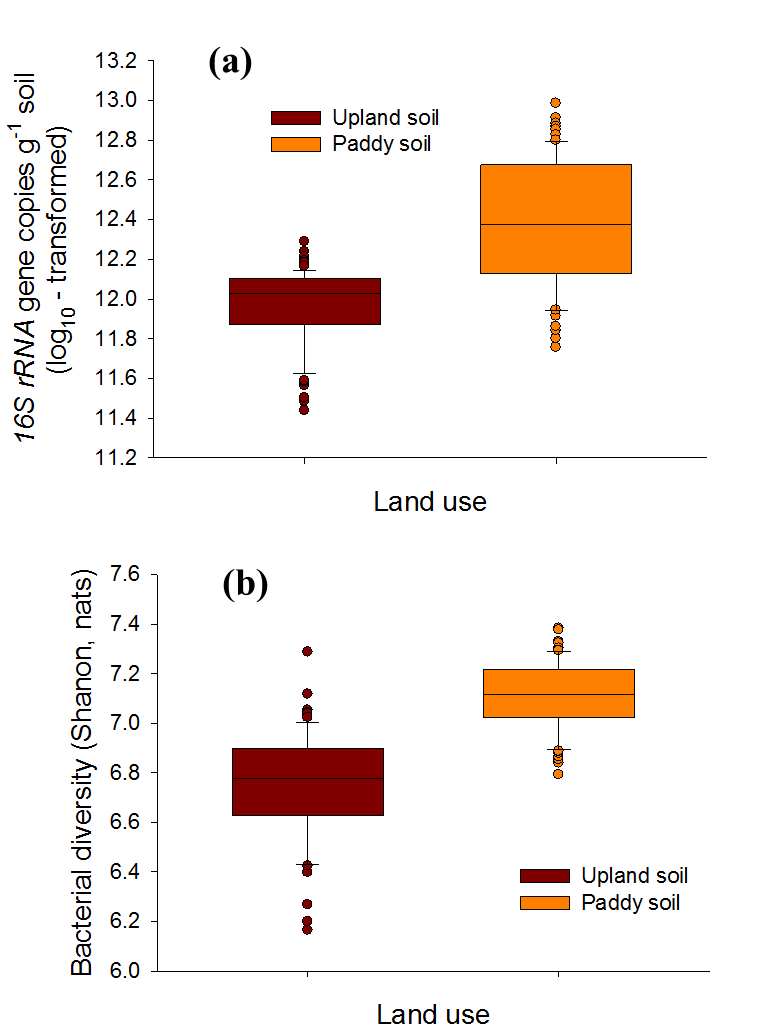


**Figure S2.** Relative mean abundances of the dominant phyla (classes) based on MiSeq sequencing of *16S rRNA* gene in paddy and upland soils.

**Figure S3.** Predictor importance of significant (*P* < 0.05) genera in *Firmicutes* (F) and *Bacteroidetes* (B) responding to soil total Hg based on Fandom Forest (RF) analyses (a). Linear relationships between the predicted main genera and soil total Hg (b). RF Importance = Increase in % mean square error.


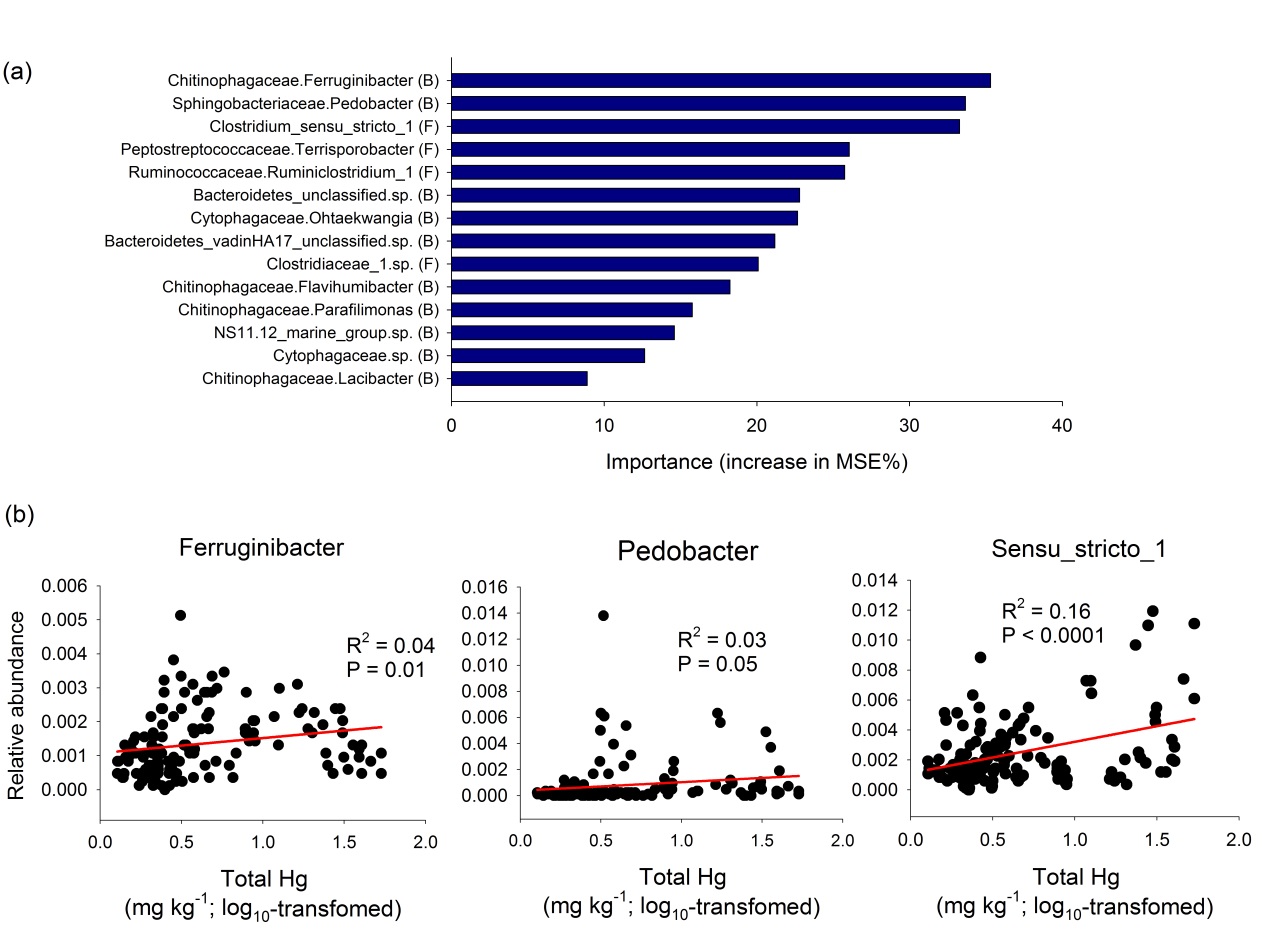


**Figure S4.** Linear relationship between the most important genus in *Nitrospirare* and soil methylmercury (MeHg) contents.

**Figure S5.** Random Forest (RF) analysis identifying the significant environmental predictors of bacterial abundance and diversity in paddy (a) and upland soils (b). RF Importance = Increase in % mean square error. Color, black and white columns represents *P* < 0.05, *P* < 0.10 and *P* > 0.10, respectively.

**Figure S6.** Random Forest (RF) analysis identifying the significant environmental predictors of the relative abundance of ecological clusters (modules #0-4) of bacterial occurrence network in paddy soils (a) and upland (b) soils. RF Importance = Increase in % mean square error. Color, black and white columns represent *P* < 0.05, *P* < 0.10 and *P* > 0.10, respectively.

**Figure S7.** Random Forest (RF) analysis identifying the significant gene predictors of soil MeHg. RF Importance = Increase in % mean square error. Only predictors from the RF with a *P* < 0.05 are selected to show in the figure.


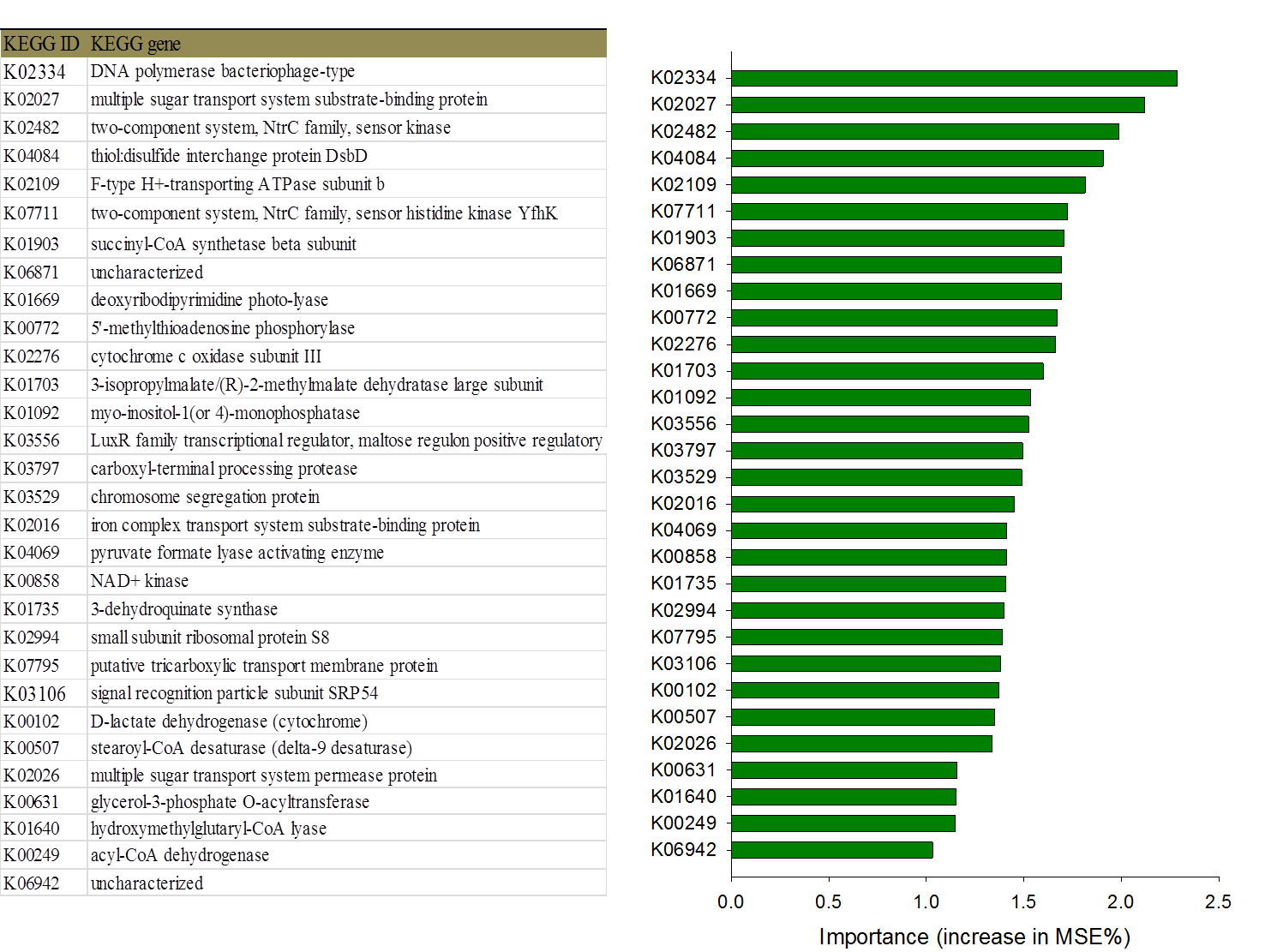


**Figure S8.** Map of the study area and 24 sampling locations around the Fenghuang (FH) and Wanshan (WS) Hg mining area across China. One paddy site and one and upland site were chosen for sampling from each of these locations. One upland site in location No. 6 was omitted because no representative upland field is adjacent to the paddy field at this location.
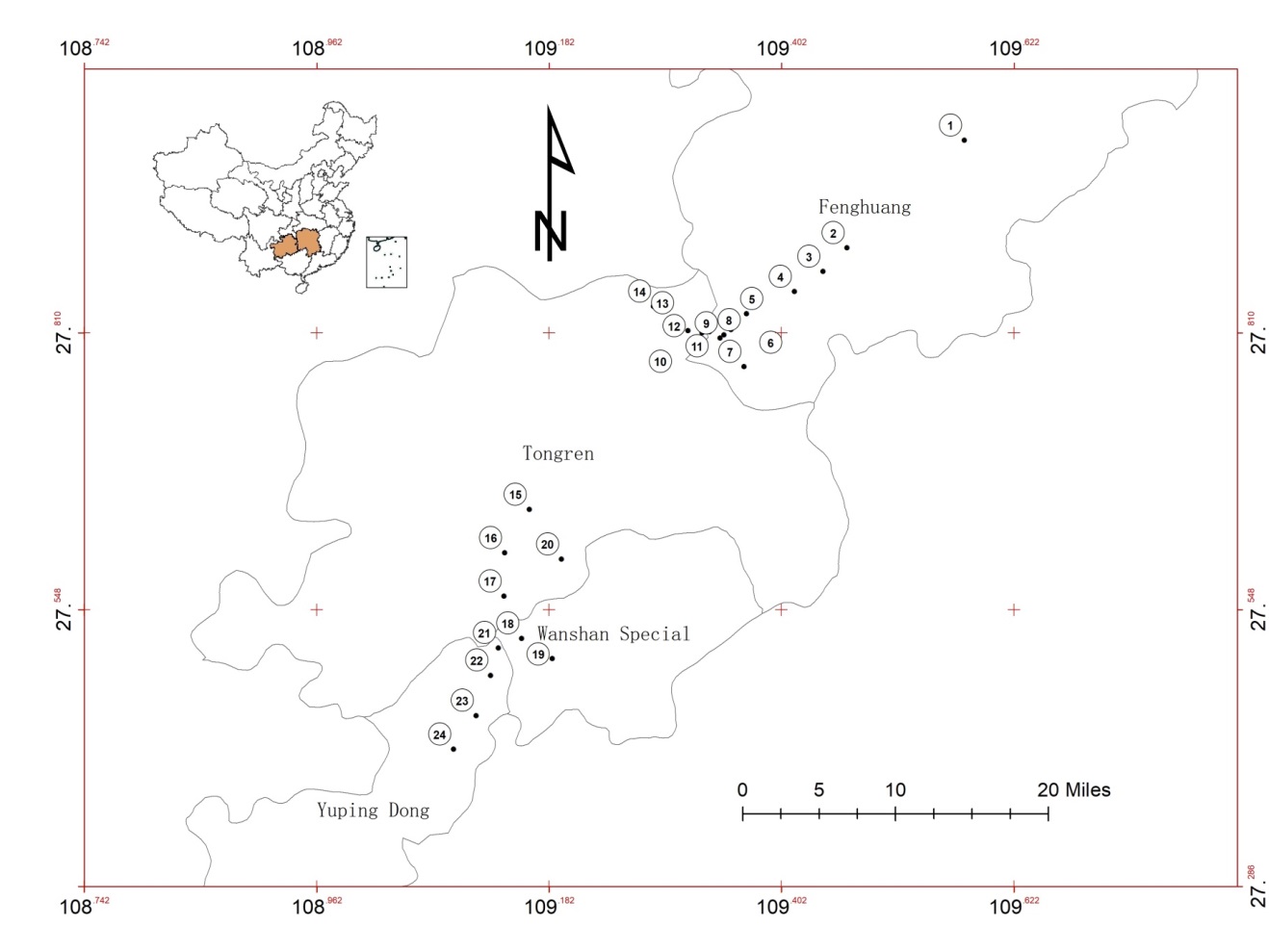


**Figure S9.** Rarefaction and Shannon curves of *16S rRNA* gene sequencing of paddy (green lines) and upland soils (red lines). The x-axis represents the number of sequences while the y-axis represents the number of detected OTU's (a) and Shannon diversity index (b), respectively.


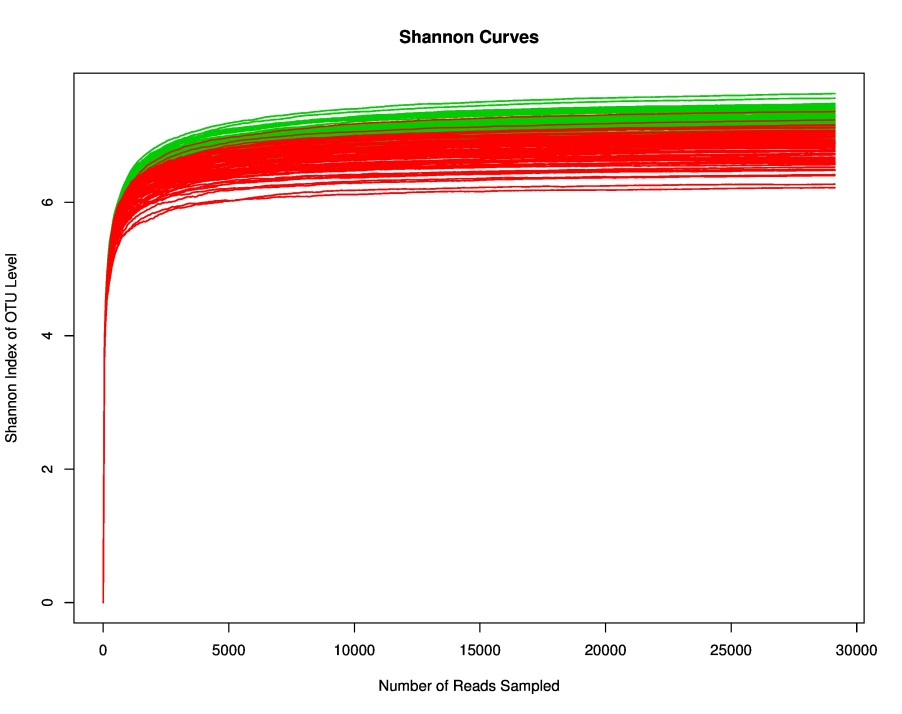

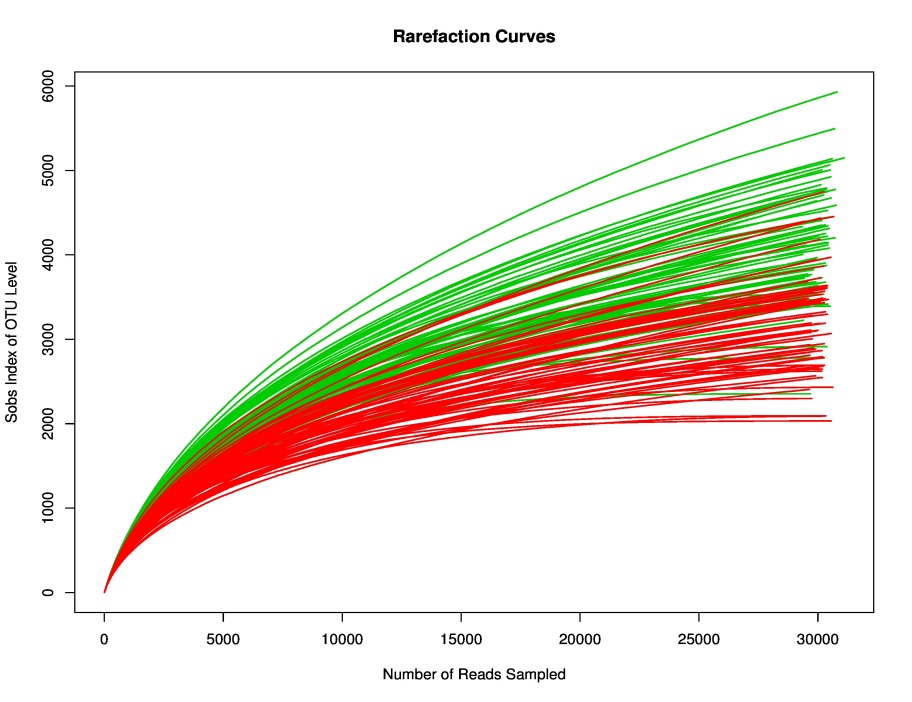


(a)

(b)

**Figure 10.** An *a priori* model identifying effects of Hg on soil abundance, diversity and the major five ecological clusters (modules, Mod) within co-occurrence network of bacterial communities. Mod#X stand for modules. The Hg box includes total Hg and methylmercury, and the spatial box includes longitude and latitude. The soil box includes soil properties that were represented by the three major components by performing principal component analysis of soil variables including pH, soil organic carbon (SOC), C: N and others (Table S3).


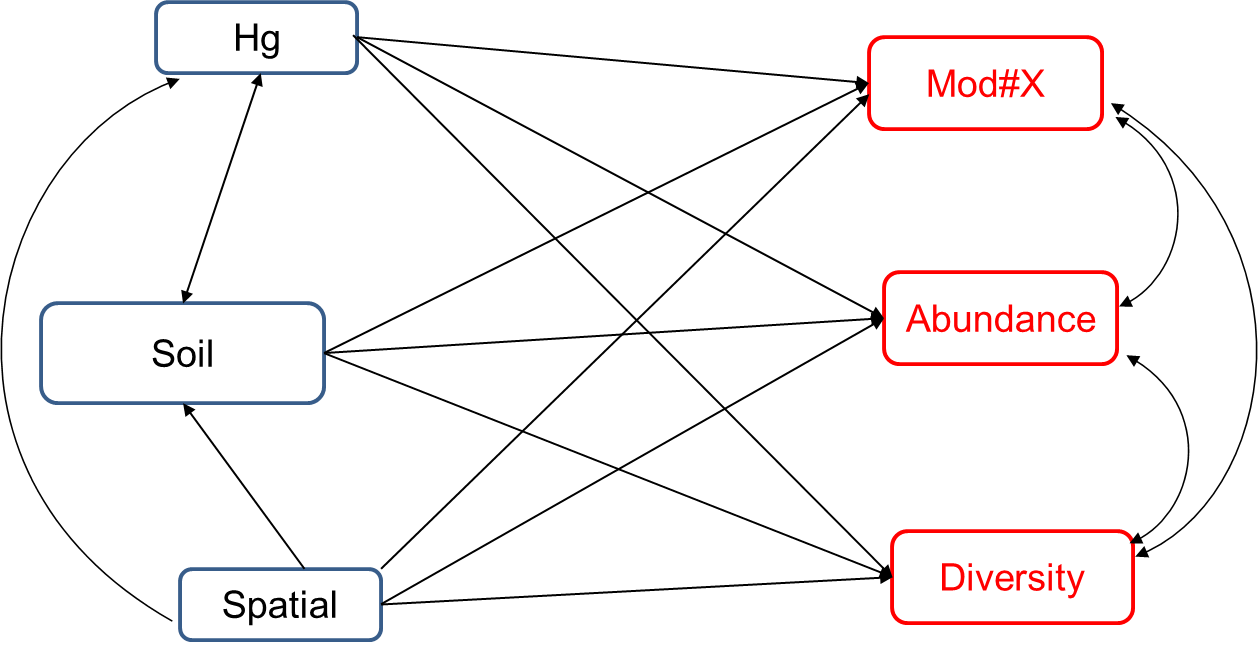

Supplement: Supplementary file 1 — Table S1. Correlation coefficients (Pearson’s ρ) between the dominant phyla/classes and soil Hg and MeHg contents. Table S2. Correlation coefficients (Spearman’s ρ) between bacterial abundance, richness and modules, and soil properties. Table S3. Correlation coefficients (Spearman’s ρ) between three major components from principal component analysis and soil properties. Table S4. Standardized direct effects from the SEM in Fig. 4. Table S5. The ID and names of the predicted functional genes (see Fig. 4) were annotated according to Kyoto Encyclopedia of Genes and Genomes (KEGG) using metagenomic data derived from a subset of our soil samples. Table S6. Correlation coefficients (Pearson’s ρ) between bacterial modules (Mod) of co-occurrence network and soil Hg and MeHg contents. Figure S1. Bacterial abundance and diversity in paddy and upland soils from Hg-impacted FH and WS areas in China Figure S2. Relative abundances of the dominant phyla (classes) based on MiSeq sequencing of 16S rRNA gene in paddy and upland soils. Figure S3. Predictor importance of main genera in Firmicutes and Bacteroideteses ponding to soil total Hg based on random forest analyses (a). Relationships between the predicted main species and soil total Hg (b). Figure S4. Relationship between the most important genus in Nitrospirare and soil MeHg. Figure S5. Random forest (RF) analyses identifying environmental predictors of soil bacterial abundance and diversity. Figure S6. Random forest analyses identifying soil environmental predictors of the relative abundance of modules #0–4 of bacterial occurrence network in paddy soils. Figure S7. Random forest analyses identifying the main significant gene predictors of soil total MeHg. Figure S8. Map of the study area and 24 sampling locations around the Hg mining area in southwest China. Figure S9. Rarefaction and Shannon curves of 16S rRNA gene sequencing of the soils. Figure S10. An a priori model identifying effects of Hg on soil microbiomes. (DOCX 1450 [file 40168_2018_572_MOESM1_ESM.docx]
