# Supplementary material for: Consistent responses of soil microbial taxonomic and functional attributes to mercury pollution across China
Source: Microbiome. 2018 Oct 18;6:183. doi: 10.1186/s40168-018-0572-7 (PMC6194565; doi:10.1186/s40168-018-0572-7)
Supplement: Supplementary file 3 — Details regarding shotgun metagenomic sequencing and gene analysis. (DOCX 18 kb) [file 40168_2018_572_MOESM3_ESM.docx]

**Shotgun metagenomic sequencing and gene analysis**

Seven paddy soils and three upland soils were selected from our 141 samples for metagenomic analysis. Genomic DNA from the soil samples were extracted in duplicate for metagenomic sequencing using the MOBiO PowerSoil kit (QIAGEN Inc., USA) according to the manufacturer's protocol. Five replicates were conducted for each sample to obtain sufficient DNA (> 1 µg) for shotgun metagenomic sequencing. Sequencing was performed using an Illumina PE150 (Illumina Inc.) at Majorbio, Inc., Shanghai, China. Raw reads (150 bp in length) were trimmed to remove low quality reads as follows. First, the SeqPrep software (<https://github.com/jstjohn/SeqPrep>) was used to remove the adapter sequences. Second, the library sickle (https://github.com/najoshi/sickle) was used to trim the reads from the 5’end to 3’end using a sliding window (size 50 bp, 1 bp step). If the mean quality of bases inside a window drops below 20, the remainder of the read below the quality threshold was trimmed. We also discarded quality-trimmed reads that were shorter than 50 bp or containing N (ambiguous bases). As a result, a total of 901,610,484 clean reads was generated with an average of 90,161,048 reads per sample. Paired reads of shotgun metagenomic sequences were merged with FLASH using default parameters ([Magoč and Salzberg, 2011](#_ENREF_41)). Using MBLASTX, merged reads were also mapped against the protein sequence of the KEGG database (E-value cutoff 1e^-6^), and the relative abundance of each KO gene was also calculated. To estimate the influence of elevated Hg and MeHg contents on these genes, we focused on the KO genes related to microbial metabolism, Hg transformations and other sensitive ones.
